# Evaluation of iodine nutritional status during pregnancy by estimated 24-h urinary iodine excretion: population variation range and individual accuracy

**DOI:** 10.1017/S1368980021003335

**Published:** 2022-02

**Authors:** Ye Bu, Yan Cai, Chunlei Ji, Chunyan Zhao, Chunyuan Tian, Bo Pang, Mengqi Shi, Xin Li, Ying Liu, Dianjun Sun

**Affiliations:** 1 Key Laboratory of Etiology and Epidemiology, National Health and Family Planning Commission, Center for Endemic Disease Control, Chinese Center for Disease Control and Prevention, Harbin Medical University, No.196, Baojian road, Harbin, Heilongjiang 150086, People’s Republic of China; 2 The Fourth Affiliated Hospital of Harbin Medical University, Department of Obstetrics and Gynecology, Harbin, Heilongjiang, People’s Republic of China; 3 Nantong Center for Disease Control and Prevention, Nantong, Jiangsu Province, People’s Republic of China

**Keywords:** Pregnant women, Iodine nutrition, Individual assessment, Urinary iodine excretion, Estimated iodine excretion, Urinary creatinine, Epidemiology study

## Abstract

**Objective:**

To explore the accuracy of estimated 24-h urinary iodine excretion (24-h UIE_est_) in assessing iodine nutritional status.

**Design:**

Fasting venous blood, 24-h and spot urine samples were collected during the day. The urinary iodine concentration (UIC) and urinary creatinine concentration (UCrC) were measured, and the urinary iodine-to-creatinine ratio (UI/Cr), 24-h UIE_est_, and 24-h urinary iodine excretion (24-h UIE) were calculated. At the population level, correlation and consistency between UIC, UI/Cr, 24-h UIE_est_ and 24-h UIE were assessed using correlation analysis and Bland–Altman plots. At the individual level, receiver operating characteristic (ROC) curves were used to analyse the accuracy of the above indicators for evaluating insufficient and excessive iodine intake. The reference interval of 24-h UIE_est_ was established based on percentile values.

**Setting:**

Indicator can accurately evaluate individual iodine nutrition during pregnancy remains controversial.

**Participants:**

Pregnant women (*n* 788).

**Results:**

Using 24-h UIE as standard, the correlation coefficients of 24-h UIE_est_ from different periods of the day ranged from 0·409 to 0·531, and the relative average differences ranged from 4·4 % to 10·9 %. For diagnosis of insufficient iodine intake, the area under the ROC curve of 24-h UIE_est_ was 0·754, sensitivity and specificity were 79·6 % and 65·4 %, respectively. For diagnosis of excessive iodine intake, the area of 24-h UIE_est_ was 0·771, sensitivity and specificity were 66·7 % and 82·0 %, respectively. The reference interval of 24-h UIE_est_ was 58·43–597·65 μg.

**Conclusions:**

Twenty-four-hour UIE_est_ can better indicate iodine nutritional status at a relatively large sample size in a given population of pregnant women. It can be used for early screening at the individual level to obtain more lead time for pregnant women.

Iodine is an indispensable trace element, and the physiological demand for iodine increases during pregnancy^(1)^. Iodine deficiency or excess has irreversible effects on fetal thyroid function, the nervous system, brain development, and the shape and function of the thyroid in pregnant women^(2,3)^. As a result, an increasing number of pregnant women have a clear clinical need for diagnostic tests to assess iodine status.

The urinary iodine concentration (UIC) has been widely used to evaluate the iodine nutritional status in pregnant women in populations. The WHO/UNICEF/ICCIDD recommended that the median UIC can be used to assess the iodine status of populations^(4)^. The Chinese guidelines on the diagnosis and management of thyroid disease during pregnancy and postpartum (2019)^(5)^ recommended that the urinary iodine-to-creatinine ratio (UI/Cr) can be used to evaluate populations’ iodine nutritional status during pregnancy. Therefore, it is still an urgent need for establishing an index that can accurately evaluate individual iodine nutrition and establishing a range of reference values.

Under the current research conditions, 24-h urinary iodine excretion (24-h UIE) is recognised as the gold standard to evaluate individuals’ recent iodine nutrition status. However, 24-h urine is difficult to collect and achieve clinical implementation and promotion, so it is necessary to find a substitute indicator to accurately estimate 24-h UIE in the field of individual iodine nutrition evaluation. In recent years, some scholars have used 24-h creatinine scaling to estimate 24-h iodine excretion in adults^(6,7)^ and children^(8,9)^. However, it is currently uncertain whether this methodological approach also provides a more accurate assessment of iodine status in pregnant women. Therefore, the purpose of this methodological study was to determine the accuracy and variation range of estimated 24-h UIE (24-h UIE_est_) in evaluating iodine nutritional status during pregnancy at both the individual and population levels.

## Materials and methods

### Study participants

From June 2018 to October 2020, pregnant women were screened in hospitals in Harbin, Heilongjiang Province, an iodine adequate area in China. The exclusion criteria were (1) clinical/subclinical thyroid dysfunction; (2) family history of thyroid disease; (3) having taken iodine-containing drugs or supplements; (4) received treatment for thyroid disease; (5) visible or palpable goitre; (6) having consumed iodine-rich foods for 4 d before providing the urine sample; (7) multiple pregnancies; (8) Fe deficiency anaemia and (9) hyperemesis gravidarum.

### Basic information collection

All participants completed questionnaires, which collected basic characteristic information, gestational history, history of taking nutritional drugs during pregnancy and disease history. In addition, we determined weight and body composition for all participants by direct segment multifrequency bioelectrical impedance analysis (DSM-BIA) with the Inbody 770 human component analyser.

### Collection and detection of urine and blood samples

Professionals gave participants detailed instructions on the correct method of collecting 24-h and spot urine samples: at 6:00 am on the first day, and participants were instructed to empty their urine. Then, in the following 24 h, urine was collected each time until 6:00 am the next day (urine at 6:00 am the next day was included). Complete urine samples were collected and placed in 3000-ml clean polyethylene barrels provided by unification. Then, participants used cylinders to measure their 24-h urine volume. After the urine was thoroughly mixed, participants used cryogenic tubes to place a 5-ml 24-h urine sample^(10)^. For 24-h urine that did not meet the above requirements, new retention was required. Pregnant women who collected 24-h urine samples were also required to collect spot urine (fasting, 8:00–9:00, 11:00–12:00 and 16:00–17:00 in 24 h) at least twice a day. We used 1·8-ml cryogenic test tubes to place spot urine samples and directly transported the urine samples to the laboratory for analysis.

UIC was determined by arsenic and cerium catalytic spectrophotometry (WS/T107-2006), and urinary creatinine concentration (UCrC) was determined by spectrophotometry (JAFF method) (WS/T97–1996). External reference samples were provided by the National Laboratory for Prevention and Treatment of Iodine-Deficient Disorders in China. We used the certified reference material (GBW9110_t_, GBW9109_l_ and GBW09108_p_) of the Center for Disease Control (CDC) of China as the control measurements. The standard values of the UIC reference materials were 239 ± 15 μg/l, 134 ± 10 μg/l and 69·5 ± 9·0 μg/l^(10)^, the interassay CV were 2·5 %, 2·2 %, and 1·7 %, and the intraassay CV were 3·1 %, 3·7 %, and 2·9 %, respectively. The target values of the urinary creatinine reference materials were 1·135 ± 0·11 g/l, the interassay CV was 3·2 % and the intraassay CV is 4·1 %.

Venous blood was collected from all pregnant women in the morning (8:00–11:00). After separating the serum, the levels of thyroid hormone (IT3000, Roche), ferritin (i2000SR, Abbott), and Hb (XN1000, Sysmex Corporation) were measured. A chemiluminescent immunoassay was used to determine the levels of thyroid hormones, including free triiodothyronine (FT3), free thyroxine (FT4), thyroid-stimulating hormone, thyroglobulin antibody and thyroid peroxidase antibody. Maternal serum ferritin levels were measured using an electrochemiluminescence immunoassay. Hb was measured using the electric resistance method.

Urine samples were preserved at 4°C, while serum samples were frozen at −20°C until analysis. All urine iodine and creatinine indicators were tested within 1 week and strictly controlled in the National Key Laboratory of Etiology and Epidemiology.

### The equation of predicted 24-h urinary creatinine excretion

Twenty-four-hour UCrE is the 24-h urinary creatinine concentration (24-h UCrC) multiplied by 24-h urine volume. Twenty-four-hour UIE_est_ is calculated by the UI/Cr multiplied by predicted 24-h UCrE. Some scholars have used UI/Cr multiplied by predicted 24-h UCrE^(11,12)^ to evaluate individual iodine nutritional status in healthy adults^(13)^. However, the equation of predicted 24-h UCrE based on age, weight, height and other factors of pregnant women has not been officially established. Therefore, the predicted 24-h UCrE we used was the result of a preliminary study by our research group^(10)^. We used the multiple linear regression method to predict 24-h UCrE based on pregnancy weight, gestational weeks, age, pre-pregnancy BMI and body fat percentage from 743 healthy pregnant women and predicted the finally constructed multiple linear regression model 24-h UCrE = 0·423 + 0·016 × pregnancy weight(kg) (*F* = 45·029, *P* < 0·001; *r*^2^ = 0·138).

### Diagnostic criteria

Using the recommended nutrient intake and tolerable upper intake levels as the standard to reversely deduce the iodine intake from the iodine excreted in the urine, Bath SC^(14)^ suggested that the median estimated 24-h UIE be >225 μg/d assuming the recommended 250 μg/d iodine intake^(4,15)^ of which 90 % is excreted^(16)^. In China, the recommended nutrient intake for iodine during pregnancy is 230 μg/d^(17)^. Therefore, in this study, 24-h UIE < 207 μg/d was defined as ‘insufficient iodine intake’. Given that the upper intake levels of iodine during pregnancy is 600 μg/d^(17)^, 24-h UIE > 540 μg/d is defined as ‘excessive iodine intake’.

The reference ranges of thyroid-stimulating hormone from different trimesters were 0·09–4·52 mU/l, 0·45–4·32 mU/l and 0·30–4·98 mU/l. The reference ranges for FT4 were 13·15–20·78 pmol/l, 9·77–18·89 pmol/l and 9·04–15·22 pmol/l, respectively. The above reference ranges were recommended by the guidelines for diagnosis and treatment of thyroid disorders in pregnancy and postpartum (2019)^(5)^. The reference ranges of FT3, thyroglobulin antibody and thyroid peroxidase antibody (IT3000, Roche) were 3·1–6·8 pmol/l, 0-115 U/ml and 0–34 U/ml throughout pregnancy, respectively.

According to the recommendation of the 2014 guidelines for the diagnosis and treatment of Fe deficiency and Fe deficiency anaemia during pregnancy^(18)^ in China, the diagnostic criteria for Fe deficiency anaemia are serum ferritin level < 20 g/l and Hb level < 110 g/l.

### Statistical analysis

We used SPSS 25. 0 (IBM) for data processing and analysis and GraphPad 8.0 software to generate statistical charts. Normally distributed data are presented as the means and standard deviation, and one-way ANOVA was used to compare means between groups. The non-normally distributed data are represented using the median with the interquartile range. The Mann–Whitney *U* rank-sum test was used for the intergroup comparison of urinary iodine indicators, and the Spearman rank correlation test was used for correlation analysis. A receiver operating characteristic (ROC) curve was established, and the area comparison test evaluation index under the ROC curve was applied. The significance levels quoted are two-sided and *P* < 0·05 was used to show statistically significant differences.

## Results

### General characteristics of pregnant women

A total of 788 pregnant women (including 208 in the first trimester, 410 in the second trimester and 170 in the third trimester) were included in this study (see online Supplemental material Fig. 1).

The baseline characteristics of pregnant women in the different trimesters are shown in Table 1. Age, current weight (when collecting 24-h urine samples), body fat percentage, fat loss weight and muscle content varied by trimester (*P* < 0·001).

**Table 1:** General characteristics of pregnant women

Group	*n*	Age		Gestational age (week^+day^)		Height (m)		Pre-pregnancy weight (kg)		Pre-pregnancy BMI (kg/m^2^)		Current weight (kg)		Body fat percentage (%)		Fat loss weight (kg)		Muscle content (kg)	
		Mean	sd			Mean	sd	Mean	sd	Mean	sd	Mean	sd	Mean	sd	Mean	sd	Mean	sd
First trimester	208	30·32	3·7	12^+6^	12, 13^+2^	1·63	0·05	59·27	10·52	22·36	3·8	60·63	11·37	27·25	6·2	43·5	4·96	40·48	5·22
Second trimester	410	30·28	3·61	18^+4^	16^+5^, 23^+5^	1·63	0·05	57·81	10·85	21·81	3·41	62·46	10·37	27·23	5·6	44·58	4·54	41·27	5·28
Third trimester	170	31·21	3·61	31	28^+6^, 33^+3^	1·63	0·05	59·07	11·83	22·32	4·24	70·42	12·18	30·15	4·81	47·82	4·82	45·49	5·17
*F*		4·182		1537·057		0·668		1·077		1·915		41·768		14·205		31·089		36·499	
*P*		0·016		<0·001		0·513		0·341		0·148		<0·001		<0·001		<0·001		<0·001	

Pre-pregnancy BMI was calculated by dividing pre-pregnancy weight by height squared.

First trimester: 0–13 weeks, second trimester: 14–27 weeks and third trimester: 28–40 weeks.

The value was presented as the means and standard deviation.

One-way ANOVA was used for the intergroup comparison, and *P* < 0 05 was considered statistically significant.

### Twenty-four-hour urinary iodine parameters, spot urinary iodine concentration and urinary iodine-to-creatinine ratio measurements

The median 24-h UIE was 205·99 μg; 188·42 μg in the first trimester, 204·85 μg in the second trimester and 232·99 μg in the third trimester, with significant differences between the trimesters (*P* < 0·01). The median 24-h UIE_est_ was 192·52 μg; 171·89 μg in the first trimester, 198·60 μg in the second trimester and 204·16 μg in the third trimester. The 24-h UIE_est_ in the first trimester was significantly different from that in the second and third trimesters (both *P* < 0·01), while the difference between the second and third trimesters was not significant (*P* > 0·05).

Spot UIC within a day had median values of 151·89 μg/l (fasting), 136·67 μg/l (8:00–9:00), 125·81 μg/l (11:00–12:00) and 130·17 μg/l (16:00–17:00). The UIC at fasting was significantly different from the UIC at the other three time points (*P* < 0·01). Spot UI/Cr had median values of 122·86 μg/g, 128·20 μg/g, 129·37 μg/g and 139·69 μg/g, for the same four time periods. However, after adjusting for spot UCrC, the spot UI/Cr at different time points detected no significant differences (*P* = 0·99).

Increasing trends were found in 24-h UIE_est_ and UI/Cr at fasting from the first to the third trimester, consistent with the changes in 24-h UIE values (*P* < 0·001). However, the median 24-h UIC between different trimesters showed no significant differences (*P* > 0·05). (see online Supplemental Material Table 1)

### Correlation analysis of spot urinary iodine concentration, urinary iodine-to-creatinine ratio and estimated 24-h urinary iodine excretion with 24-h urinary iodine excretion

**Fig. 1:**
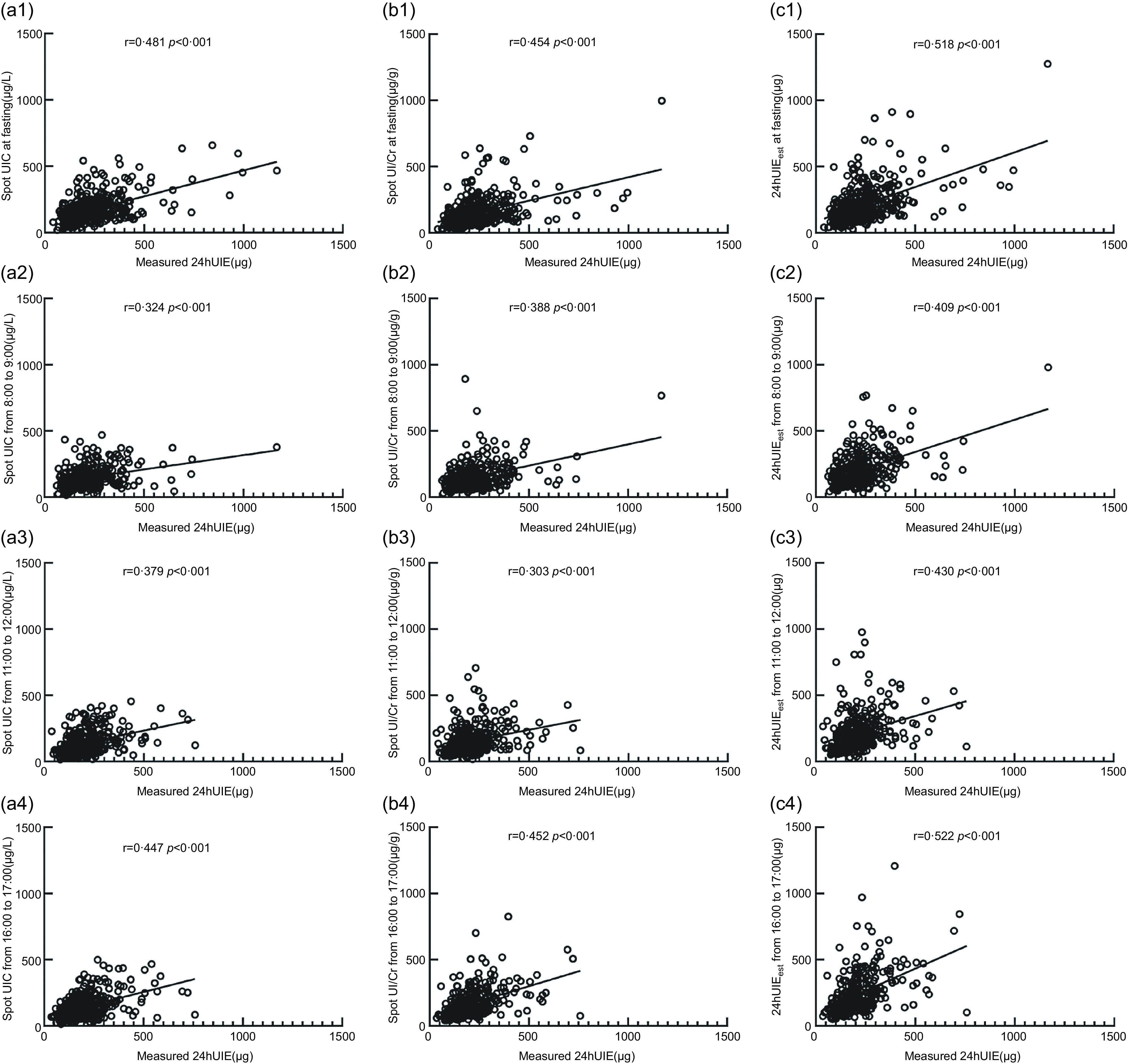
Correlation between spot UIC, UI/Cr, 24-h UIE_est_ and 24-h UIE. *UIC* urinary iodine concentration, UI/Cr, urinary iodine-to-creatinine ratio; 24-h UIE_est_; estimated 24-h urinary iodine excretion; 24-h UIE, 24-h urinary iodine excretion. a, b and c represent the consistency of spot UIC, UI/Cr and 24-h UIE_est_ compared with 24-h UIE, respectively. The urinary indicators at four time periods (at fasting, 8:00–9:00, 11:00–12:00 and 16:00–17:00) are expressed by tail 1, tail 2, tail 3 and tail 4

Compared with 24-h UIE measurements, the correlation coefficients for spot UIC ranged from 0·324 to 0·481 (*P* < 0·01), those for UI/Cr were 0·303–0·454 (*P* < 0·01), and those for 24-h UIE_est_ were 0·409–0·531 (*P* < 0·01). The correlation coefficients of urinary indicators at fasting were higher than those at other time points of the day. Moreover, the correlation coefficients for 24-h UIE_est_ at different time points within a day were higher than the other two indexes for the corresponding time points (Fig. 1).

### Consistency of urinary iodine concentration, urinary iodine-to-creatinine ratio and estimated 24-h urinary iodine excretion with 24-h urinary iodine excretion at each time point

**Fig. 2:**
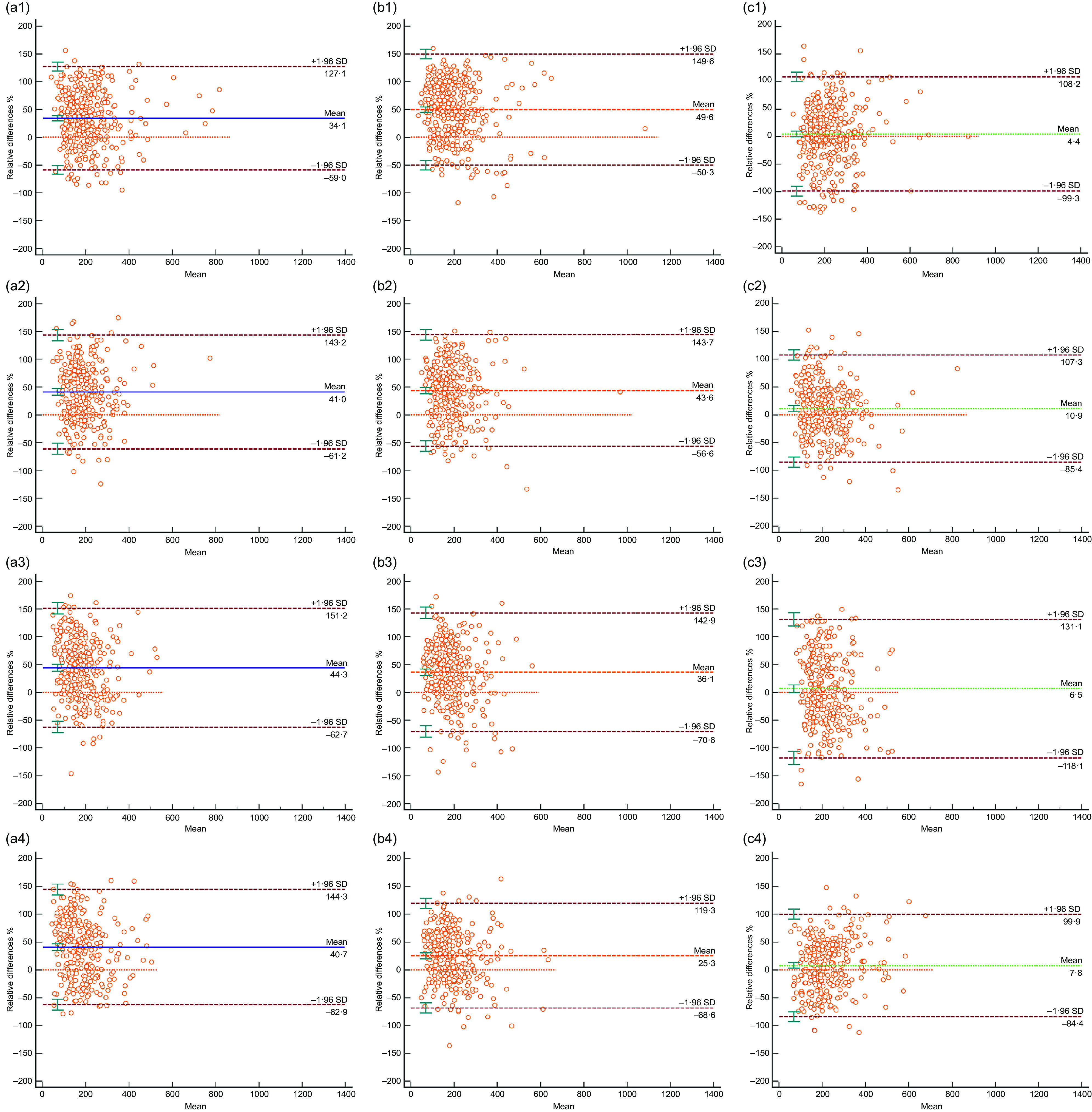
Consistency of spot UIC, UI/Cr and 24-h UIE_est_ compared with 24-h UIE at different urine collection times. UIC, urinary iodine concentration; UI/Cr, urinary iodine-to-creatinine ratio; 24-h UIEest, estimated 24-h urinary iodine excretion; 24-h UIE, 24-h urinary iodine excretion; *n*, number of participants. The Bland–Altman plot shows the consistency of spot UIC (a), UI/Cr (b) and 24-h UIE_est_ (c) compared with 24-h UIE. The urinary indicators at four time periods (at fasting, 8:00–9:00, 11:00–12:00 and 16:00–17:00) are expressed by tail 1, tail 2, tail 3 and tail 4. The solid line represents the relative average difference, and the dotted line represents the 95 % reference interval for the relative average difference. On the X-axis, ‘Mean’ means the average of spot UIC, UI/Cr or 24-h UIE_est_ with 24-h UIE

Using 24-h UIE as the gold standard, we found that the relative average differences in 24-h UIE_est_ between different periods during pregnancy clearly distinguished UIC and UI/Cr for the same periods. The relative average difference in 24-h UIE_est_ ranged from 4·4 % to 10·9 % for the different time periods, while that for UIC and UI/Cr ranged from 34·1 % to 44·3 % and 25·3 % to 49·6 %, respectively (Fig. 2).

### ROC curve

**Fig. 3:**
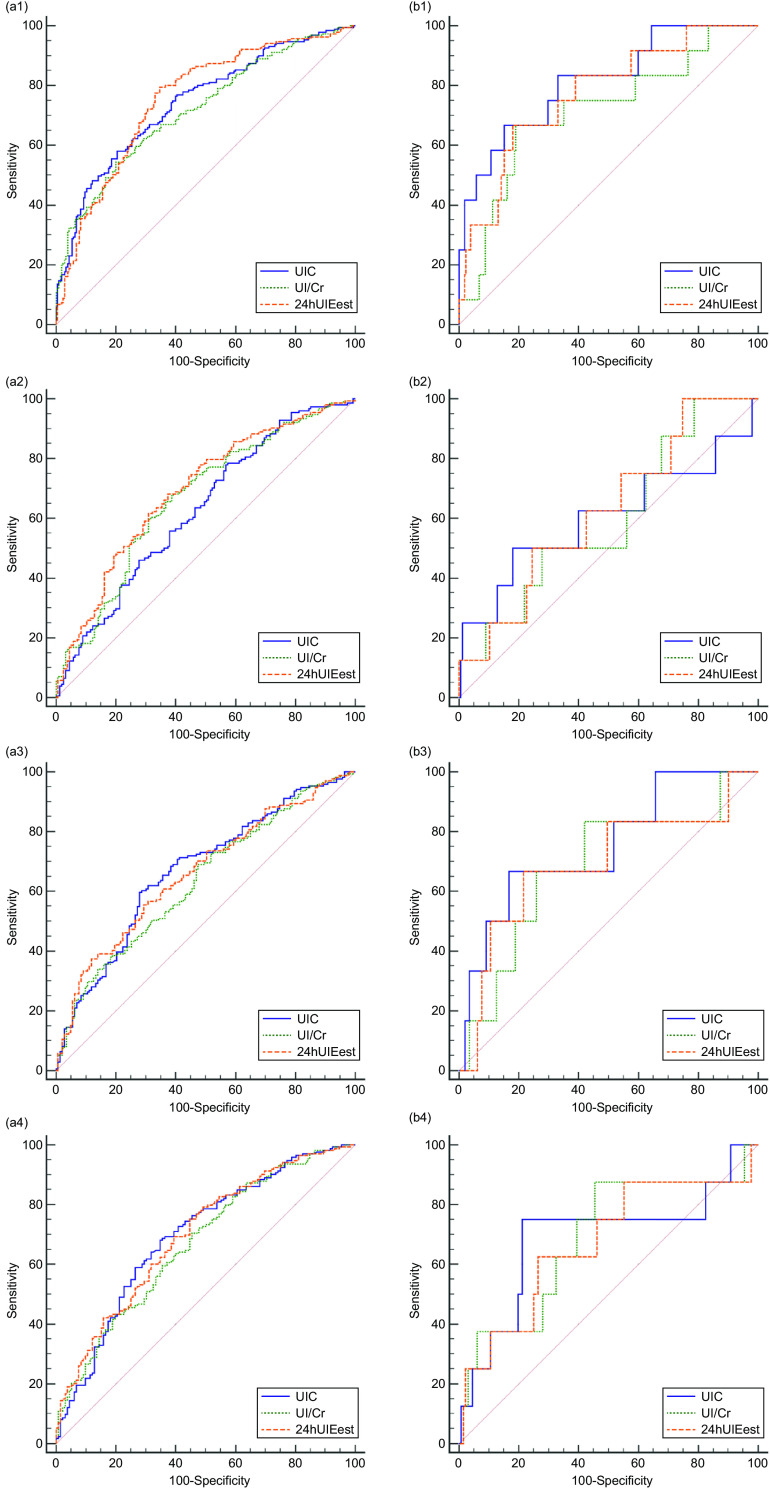
ROC curves and tangent point values for UIC, UI/Cr and 24-h UIE_est_ from different urine collection times. UIC, urinary iodine concentration; UI/Cr, urinary iodine-to-creatinine ratio; 24-h UIE_est_, estimated 24-h urinary iodine excretion; 24-h UIE, 24-h urinary iodine excretion; *n*, number of participants. a and b represent the ROC curves of UIC, UI/Cr and 24-h UIE_est_ for diagnosing insufficient iodine intake and excessive iodine intake, respectively. The urinary indicators at four time periods (at fasting, 8:00–9:00, 11:00–12:00 and 16:00–17:00) are expressed by tail 1, tail 2, tail 3 and tail 4

ROC curves of UIC, UI/Cr and 24-h UIE_est_ at different times were established using these indicators as the gold standard (Fig. 3). Either UIC, UI/Cr or 24-h UIE_est_, when diagnosed with insufficient iodine intake or excessive iodine intake, had the AUC as the highest at fasting compared with the other three time points. For the diagnosis of insufficient or excessive iodine intake, the AUC of 24-h UIE_est_ showed no difference from UIC at fasting; however, there were differences with UI/Cr at fasting with *P*-values of 0·007 and 0·033, respectively. In general, regardless of which urinary indicator was used for the diagnosis of insufficient iodine intake, the sensitivity ranged from 54·5 % to 79·6 %, and the specificity ranged from 42·6 % to 80·0 %. For the diagnosis of excessive iodine intake, the sensitivity ranged from 50 % to 87·5 %, and the specificity ranged from 54·5 % to 84·9 % (Table 2).

**Table 2:** Receiver operating characteristic (ROC) curves of insufficient and excessive iodine intake

	Insufficient iodine intake						Excessive iodine intake					
	UIC		UI/Cr		24-h UIE_est_		UIC		UI/Cr		24-h UIE_est_	
The AUC (95 % CI)												
At fasting	0·743	0·697, 0·785	0·721	0·674, 0·765	0·754 †	0·708, 0·795	0·814	0·756, 0·864	0·714	0·649, 0·773	0·771 †	0·709, 0·825
8:00–9:00	0·627	0·571, 0·681	0·667	0·611, 0·719	0·691*,†	0·637, 0·743	0·602	0·522, 0·677	0·595	0·516, 0·671	0·625	0·546, 0·699
11:00–12:00	0·67	0·615, 0·722	0·636	0·58, 0·689	0·663†	0·608, 0·715	0·752	0·674, 0·819	0·683	0·602, 0·757	0·690	0·609, 0·763
16:00–17:00	0·697	0·642, 0·748	0·672	0·616, 0·724	0·698†	0·643, 0·749	0·687	0·603, 0·762	0·688	0·604, 0·763	0·669	0·584, 0·746
Sensitivity												
At fasting	58·1		54·5		79·6		66·7		66·7		66·7	
8:00–9:00	78·6		68·2		68·2		50		50		50	
11:00–12:00	59·6		69		55·6		66·7		83·3		66·7	
16:00–17:00	68·2		70·5		75·1		75		87·5		62·5	
Specificity												
At fasting	79·5		80·0		65·4		84·9		81·0		82	
8:00–9:00	42·6		60·6		62·6		81·9		72·3		75·5	
11:00–12:00	72		52·4		70·6		83·2		58		78·3	
16:00–17:00	65·2		54·5		55·3		79·1		54·5		73·5	

UIC, urinary iodine concentration; UI/Cr, urinary iodine-to-creatinine ratio; 24-h UIE_est_, estimated 24-hour urinary iodine excretion.

* Compared with UIC at the same time periods, *P* < 0 05.

† Compared with UI/Cr at the same time periods, *P* < 0 05.

### Reference values of 24-h urinary iodine excretion, estimated 24-h urinary iodine excretion (based on fasting urinary iodine-to-creatinine ratio), urinary iodine concentration and urinary iodine-to-creatinine ratio at fasting

We established the reference intervals of urinary iodine indicators for healthy pregnant women based on percentile values (Table 3). The reference interval of 24-h UIE was 78·95–553·29 µg. The reference intervals of 24-h UIE_est_, UIC and UI/Cr at fasting were 58·43–597·65 μg, 42·93–451·33 μg/l and 37·54–509·28 μg/g), respectively.

**Table 3:** Reference values of 24-h UIE, 24-h UIE_est_ (based on fasting UI/Cr), UIC and UI/Cr at fasting during the entire pregnancy and for each trimester

Indicator	Pregnancy(in total)	First trimester	Second trimester	Third trimester
24-h UIE (μg)	78·95–553·29	73·60–666·83	77·77–530·58	90·86–555·36
24-h UIE_est_ (μg)	58·43–597·65	47·37–474·84	64·63–648·87	70·13–762·50
Spot UIC (μg/l)	42·93–451·33	28·87–608·36	42·02–462·62	69·50–420·28
Spot UI/Cr (μg/g)	37·54–509·28	31·54–315·43	39·665–548·05	42·17–618·80

24-h UIE, 24-hour urinary iodine excretion; 24-h UIE_est_, estimated 24-hour urinary iodine excretion; UIC, urinary iodine concentration; UI/Cr, urinary iodine-to-creatinine ratio.

First trimester: 0–13 weeks, second trimester: 14–27 weeks and third trimester: 28–40 weeks.

Described as the median, 2 5th and 97 5th centiles (P_2 5_–P_97 5_).

## Discussion

Iodine is an essential trace element for synthesising thyroid hormone, which is essential for human growth and metabolism. Both the WHO/UNICEF/ICCIDD and Chinese Medical Association (CMA) recommend that pregnant women increase their nutritional iodine intake per day^(4,17)^. At present, urinary iodine indicators are generally used to evaluate iodine nutrition at the population level. However, at the individual level, it is still necessary to find a more accurate biomarker and confirm its reference interval. Therefore, we conducted a systematic and comprehensive methodological study on urinary iodine indicators.

Our study demonstrated that spot UIC at fasting of 788 pregnant women varied by the other three time points of the day (*P* < 0·001). This may be due to the hydration status and circadian rhythmicity during pregnancy. However, after adjusting for spot UCrC, the differences in UI/Cr were not significant. This further proved that the influence of urine volume on UIC could be excluded after creatinine correction. In different trimesters, the variation trend of 24-h UIC was different from that of 24-h UIE, which was consistent with 24-h UIE_est_. Spot UIC at the same time points also showed no obvious change rule (*P* > 0·05). The variation in spot UIC at fasting was different from the result of Pan^(19)^; however, the variation in UI/Cr was consistent with Pan. Theoretically, with the increase in gestational weeks, morning sickness gradually subsides, and the total dietary energy intake and renal clearance of iodine increase, all of which caused 24-h UIE to show a gradually increasing trend^(20)^. Although it has previously been shown that UIC is unstable in children and adults^(21,22)^, our study further proves that UIC during pregnancy is unstable at different time periods of the day and in different trimesters.

In recent years, in the study of urine indicators, many investigators have used creatinine to correct UIC in a one-time random urine sample^(23,24)^. However, few studies have focused on urinary creatinine metabolism during pregnancy^(25)^. Our previous research demonstrated that spot UCrC varied irregularly during different trimesters and at different time periods of the day^(10)^. Although creatinine is known to be excreted at a relatively constant rate in 24 h^(26)^, spot UCrC are still affected by urine volume and circadian rhythmicity^(8)^. Furthermore, weight growth is evident during pregnancy, including not only the pregnant woman’s own increase in blood volume, breast, fat, uterus and fluid, but also the fetus, placenta, amniotic fluid and other weight. In addition to pregnancy-specific physiological changes (such as an increased glomerular filtration rate)^(27)^ and increased dietary protein intake^(28)^, the urine creatinine output during pregnancy is inevitably different from that during non-pregnancy. Our preliminary study results confirmed that pregnancy weight was an influencing factor on 24-h UCrE after adjusting for gestational weeks, age, pre-pregnancy BMI and percentage of body fat^(10)^. Thus, we established a multiple regression equation for 24-h UCrE in healthy pregnant women and made a comprehensive comparison of the relationship between UIC, UI/Cr, estimated 24-h UIE (24-h UIE_est_) and 24-h UIE.

Judging from our data, the correlation analysis found that in different periods of pregnancy, the UIC, UI/Cr and 24-h UIE_est_ all showed moderate correlations with the 24-h UIE (Fig. 1), which was consistent with previous studies of Perrine CG^(13)^. The consistency analysis showed that the relative average difference of 24-h UIE_est_ ranged from 4·4 % to 10·9 %, which was better than those of UIC and UI/Cr as well as consistent with previous studies of Liu XB^(29)^. Furthermore, the relative average difference of 24-h UIE_est_ (based on UI/Cr at fasting) was the lowest. Therefore, UIC, UI/Cr or 24-h UIE_est_ could theoretically evaluate the iodine nutritional status within a given population of pregnant women. However, 24-h UIE_est_ (based on UI/Cr at fasting) is the best estimate of 24-h UIE compared to the other two indicators.

Based on the above results at the population level, we drew the ROC curves of UIC, UI/Cr and 24-h UIE_est_. To diagnose insufficient iodine intake and excessive iodine intake, the AUC of UIC, UI/Cr and 24-h UIE_est_ at fasting were highest compared with the other three time periods within groups. The AUC of UI/Cr at fasting had significant differences with 24-h UIE_est_ at fasting, indicating that 24-h UIE_est_ may be better than UI/Cr for the diagnosis of insufficient or excessive iodine intake in diagnostic efficiency. Although there was no difference between the AUC of UIC and 24-h UIE_est_ at fasting, the correlation coefficient for spot UIC at fasting was lower than 24-h UIE_est_ at the same time period. Furthermore, the relative average difference in spot UIC at fasting was higher than 24-h UIE_est_, thereby indicating that the strength of 24-h UIE_est_ as a marker of individual iodine nutritional status was better than spot UIC and UI/Cr^(30)^. However, the gold standard we chose was 24-h UIE, for which diagnostic criteria were defined according to the iodine recommended nutrient intake and upper intake levels. Choosing different indicators as the gold standard may lead to different results, so the results of ROC curves might not be consistent with Li^(31)^.

Sensitivity and specificity reflected the screening efficiency of urinary indicators in evaluating iodine nutrition^(32,33)^. The author considers that the main factor affecting the consistency is the biological variation of the subject. Due to the significant variation of spot urinary indicators of the individual^(34)^, the same subject’s clinical measurement value at different times fluctuates. Pregnancy is a unique physiological period, when as the uterus gradually increases in size, the bladder pressure and hormone levels also increase, leading to an increased frequency of urination. As a result, the volume of every spot of urine in a day varies, affecting the spot UIC^(35)^ (see online Supplemental material Table 1).

Compared with urinary indicators, blood indicators such as serum iodine, thyroglobulin or thyroid-stimulating hormone do not change immediately with changes in dietary iodine intake^(36–39)^. Because there are generally 9 months for pregnant women from the discovery of pregnancy to full-term delivery, missing iodine-deficient pregnant women might miss the best period of iodine supplementation^(40)^, leading to adverse pregnancy outcomes and consequences. It can be hypothesised that to evaluate the short-term iodine intake of pregnant women, the blood indicators may lag behind urinary iodine indicators in time. Therefore, it is undeniable that the urine index is still of significance in evaluating individual iodine nutrition in pregnant women. Finally, we established the reference values of 24-h UIE, 24-h UIE_est_ (based on UI/Cr and 24-h UCrE_est_), UIC and UI/Cr at fasting throughout pregnancy and each trimester. The trend of the 95 % reference interval of the urinary indicators for healthy pregnant women was consistent with Liu^(41)^ and Wang^(42)^.

The author believes that future research should focus on the following aspects: first, to the best of our knowledge, very few extensive sample studies have focused on the metabolic rule of urinary creatinine. Reference values or multiple linear regression equations for 24-h UCrE in healthy pregnant women in different regions should be established. Second, it is necessary to research the relationship between the duration of iodine deficiency or excess state and the changes in haematological indicators related to iodine nutrition. Further research could be associated with serum iodine, thyroglobulin, thyroid-stimulating hormone of maternal or newborn, FT4, ferritin, or selenium for the combined screening test to improve sensitivity and specificity. Last, in the field of iodine nutrition during pregnancy, research mainly focuses on maternal iodine nutrition status and intellectual and nervous system development, IQ and executive function, attention-deficit hyperactivity disorder or autistic traits, and language skills of the offspring^(43,44)^. However, it is not scientific to use only the WHO-endorsed cut-off of less than 150 µg/l from spot UIC or UI/Cr to evaluate maternal iodine nutritional status and then compare it with the neural and intellectual development of offspring, as the criteria for individual iodine deficiency are still controversial^(32)^. Supposing individual criteria can be found, a reference range is proposed. In that case, the reference range may provide better criteria for determining individual iodine deficiency and iodine excess in the above studies, thus further promoting research progress in the field of iodine nutrition in pregnancy.

In conclusion, 24-h UCrE has an essential application in the assessment of individual iodine nutrition. We can use 24-h UIE_est_ for early screening to obtain more lead time for pregnant women.

### Strengths and limitations

#### Strengths

(1) To the best of our knowledge, this was a large-scale population cross-sectional study on the iodine nutrition evaluation method for pregnant women based on 24-h urine and (2) there have been few reports on the 24-h creatinine metabolism of healthy pregnant women in recent decades, which limited the use of 24-h UIE_est_ in a summary of iodine nutrition evaluation during pregnancy. Therefore, we established a multiple linear regression equation of 24-h UCrE according to our previous research data to obtain 24-h UIE_est_; and (3) we also measured four spot UIC during the day to verify that UIC changed significantly in 1 d.

#### Limitations

(1) The sample size of pregnant women in each trimester was not uniformly distributed; (2) this was not a longitudinal cohort study; (3) not every pregnant woman collected urine at four different time points of the day; (4) we did not conduct a dietary survey to calculate dietary iodine intake accurately; and (5) we did not include blood indicators for analysis, and our follow-up studies are ongoing.

## Supplementary material

For supplementary material accompanying this paper visit https://doi.org/10.1017/S1368980021003335. click here to view supplementary material
