# Supplementary material for: Evaluation of iodine nutritional status during pregnancy by estimated 24-h urinary iodine excretion: population variation range and individual accuracy
Source: Public Health Nutr. 2021 Aug 12;25(2):237–47. doi: 10.1017/S1368980021003335 (PMC8883787; doi:10.1017/S1368980021003335)
Supplement: Supplementary file 1 [file S1368980021003335sup.zip › S1368980021003335sup001.docx]

**Supplementary material Table**

**Supplementary material Table 1** 24hUIE, 24hUIC, 24hUIE_est_, spot UIC and UI/Cr for the whole pregnancy and for the different trimesters

| Group | n | 24hUIE(µg) | 24hUIC（µg/L） | 24hUIE_est_(µg) | spot urine | |
| --- | --- | --- | --- | --- | --- | --- |
|  |  |  |  |  | UIC（µg/L） | UI/Cr（µg/g） |
| Pregnancy in total | 788 | 205.99  (151.38,272.28) | 117.51  (86.21,165.51) | 192.52  (129.58,285.27) |  |  |
| Fasting | 408 | 216.75  (156.04,287.12) | 123.33  (87.54,173.72) | 202.27  (136.97,294.64) | 151.89^‡,§,\|\|^  (105.74,209.89) | 122.86  (82.50,189.83) |
| 8:00-9:00 | 317 | 210.56  (153.18,270.56) | 119.61  (87.32,172.15) | 179.67  (129.00,274.38) | 136.67  (92.83,186.93) | 128.20  (90.03,186.63) |
| 11:00-12:00 | 320 | 201.37  (147.14,265.85) | 113.29  (86.26,164.27) | 193.57  (131.32,297.03) | 125.81  (84.98,185.65) | 129.37  (86.04,201.77) |
| 16:00-17:00 | 313 | 199.92  (142.60,264.50) | 113.15  (82.82,164.24) | 209.65  (139.90,307.75) | 130.17  (76.40,200.01) | 139.69  (100.60,219.53) |
| First trimester | 208 | 188.42^†^  (131.45,250.32) | 114.72  (86.98,165.79) | 171.89^*,†^  (113.31,250.89) |  |  |
| Fasting | 106 | 192.58  (130.38,258.97) | 120.34  (86.54,166.28) | 145.66  (98.03,189.87) | 146.83  (96.92,189.77) | 89.30  (63.30,141.27) |
| 8:00-9:00 | 66 | 198.65  (129.66,259.04) | 115.18  (88.02,153.43) | 128.23  (98.42,190.25) | 130.45  (80.95,184.66) | 100.79  (70.11,140.67) |
| 11:00-12:00 | 89 | 185.67  (147.85,249.76) | 113.43  (92.24,170.69) | 198.41  (115.55,264.31) | 127.94  (74.43,211.34) | 131.88  (85.90,196.39) |
| 16:00-17:00 | 84 | 184.27  (137.62,250.64) | 114.52  (90.03,177.26) | 221.11  (134.07,325.55) | 140.95  (84.36,206.88) | 163.88  (96.96,237.46) |
| Second trimester | 410 | 204.85^†^  (148.82,269.49) | 114.55  (84.22,161.85) | 198.60  (137.28,291.39) |  |  |
| Fasting | 208 | 210.95  (149.03,282.07) | 118.45  (84.96,174.78) | 214.25  (145.90,290.36) | 152.63  (106.48,218.56) | 133.12  (95.13,191.26) |
| 8:00-9:00 | 183 | 204.49  (146.98,267.41) | 115.39  (82.65,174.21) | 186.38  (135.36,275.78) | 137.81  (95.65,188.13) | 134.82  (96.76,187.88) |
| 11:00-12:00 | 160 | 205.69  (147.80,265.99) | 120.25  (89.24,163.52) | 188.58  (128.16,309.63) | 128.67  (86.29,184.28) | 136.02  (91.73,216.57) |
| 16:00-17:00 | 167 | 198.65  (147.09,255.60) | 112.85  (83.12,155.24) | 204.60  (140.36,304.43) | 124.03  (74.18,187.91) | 139.06  (104.89,220.83) |
| Third trimester | 170 | 232.99  (171.33,296.90) | 124.75  (89.14,184.21) | 204.16  (131.70,317.15) |  |  |
| Fasting | 94 | 255.92  (180.55,338.34) | 143.96  (105.74,193.64) | 253.10  (149.32,384.98) | 153.81  (113.97,210.98) | 134.56  (104.75,209.02) |
| 8:00-9:00 | 68 | 232.99  (178.75,310.16) | 142.74  (103.48,192.09) | 220.00  (136.13,317.97) | 135.70  (94.07,187.20) | 138.11  (94.77,233.82) |
| 11:00-12:00 | 71 | 207.98  (142.81,282.35) | 101.28  (72.74,156.86) | 172.03  (111.18,239.25) | 110.98  (84.65,153.82) | 110.82  (73.54,162.01) |
| 16:00-17:00 | 62 | 207.04  (135.92,283.83) | 110.32  (76.22,163.42) | 195.97  (137.52,288.46) | 136.01  (64.18,226.55) | 127.67  (94.69,193.79) |

*24hUIC* 24-hour urinary iodine concentration, *24hUIE* 24-hour urinary iodine excretion, *24hUIE_est_* estimated 24-hour urinary iodine excretion, *24hUI/Cr* 24-hour urinary iodine-to-creatinine ratio, *UIC* urinary iodine concentration, *UI/Cr* urinary iodine-to-creatinine ratio. *First trimester*: 0-13 weeks, *second trimester*: 14-27 weeks, *third trimester*: 28-40 weeks. ^*^ compared with the second trimester, *p*＜0.01. ^†^, compared with the third trimester, *p*＜0.01. ^‡^，compared with UIC at 8:00-9:00, *p*＜0.01. ^§^, compared with UIC at 11:00-12:00, *p*＜0.01. ^||^, compared with UIC at 16:00-17:00, *p*＜0.01. The value were describe by median with interquartile range[M(IQR)].
