# Supplementary figures and images for: Evaluation of iodine nutritional status during pregnancy by estimated 24-h urinary iodine excretion: population variation range and individual accuracy
Source: Public Health Nutr. 2021 Aug 12;25(2):237–47. doi: 10.1017/S1368980021003335 (PMC8883787; doi:10.1017/S1368980021003335)

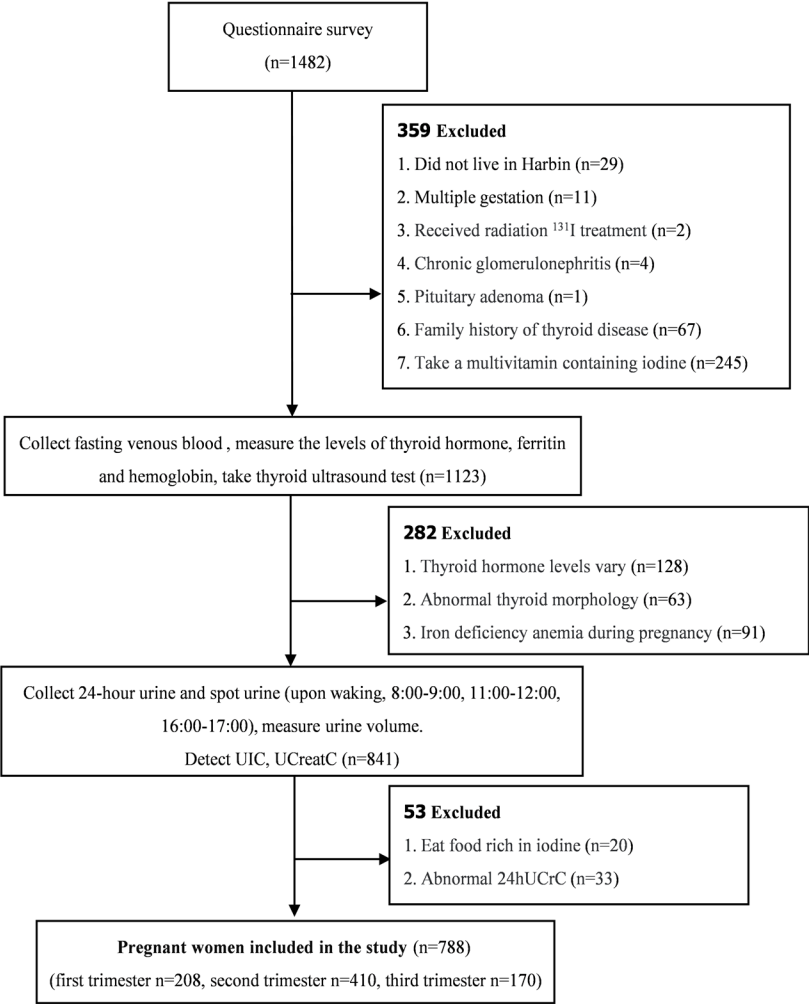

Figure 1. Participant screening process

Supplement: Supplementary file 1 [file S1368980021003335sup.zip › S1368980021003335sup002.pdf]
